# Synthesis of a MoS*_x_*–O–PtO*_x_* Electrocatalyst with High Hydrogen Evolution Activity Using a Sacrificial Counter‐Electrode

**DOI:** 10.1002/advs.201801663

**Published:** 2019-01-12

**Authors:** Yingxin Zhan, Yi Li, Zhi Yang, Xiongwei Wu, Mengzhan Ge, Xuemei Zhou, Junjie Hou, Xiannuo Zheng, Yuchong Lai, Rongrong Pang, Huan Duan, Xi'an Chen, Huagui Nie, Shaoming Huang

**Affiliations:** ^1^ Nanomaterials & Chemistry Key Laboratory Wenzhou University Wenzhou 325027 China; ^2^ College of Science Hunan Agricultural University Changsha Hunan 410128 China; ^3^ School of Material and Energy Guangdong University of Technology Guangzhou 510006 China; ^4^ School of Chemistry and Chemical Engineering Southwest University Chongqing 400715 China

**Keywords:** hydrogen evolution reaction, MoS_x_, O substitution, PtO_x_, sacrificial‐counter‐electrodes

## Abstract

Water splitting is considered to be a very promising alternative to greenly produce hydrogen, and the key to optimizing this process is the development of suitable electrocatalysts. Here, a sacrificial‐counter‐electrode method to synthesize a MoS*_x_*/carbon nanotubes/Pt catalyst (0.55 wt% Pt loading) is developed, which exhibits a low overpotential of 25 mV at a current density of 10 mA cm^−2^, a low Tafel slope of 27 mV dec^−1^, and excellent stability under acidic conditions. The theory calculations and experimental results confirm the high hydrogen evolution activity that is likely due to the fact that the S atoms in MoS*_x_* can be substituted with O atoms during a potential cycling process when using Pt as a counter‐electrode, where the O atoms act as bridges between the catalytic PtO*_x_* particles and the MoS*_x_* support to generate a MoS*_x_*–O–PtO*_x_* structure, allowing the Pt atoms to donate more electrons thus facilitating the hydrogen evolution reaction process.

## 1 Introduction

The mitigation of modern day concerns regarding a serious energy crisis, air pollution, and global warming urgently requires a clean, renewable energy source to replace traditional fossil fuels.^1^, ^2^ Hydrogen has become a preferred alternative energy source because its combustion products are pollution‐free. At present, hydrogen is prepared primarily through steam methane, petroleum cracking, and water gas conversion processes.^1^, ^2^, ^3^, ^4^ However, it is obvious that these methods are unsustainable and will eventually result in additional environmental pollution. Water electrolysis is considered to be a very promising alternative, and the key to optimizing this process is the development of suitable electrocatalysts.^5^, ^6^, ^7^, ^8^ To date, the most effective electrocatalysts for the hydrogen evolution reaction (HER) are Pt and its alloys, but the high cost of Pt remains an obstacle to its widespread application.^9^, ^10^ Although there have been recent reports of nonnoble metal compounds (including MoS_2_,^11^, ^12^, ^13^, ^14^ CoP,^15^, ^16^, ^17^ and WC^18^, ^19^) and hybrid catalysts with low Pt loadings (such as TiO_2_–Pt,^20^, ^21^ CoP–Pt,^22^ and WC‐Pt^23^), to the best of our knowledge, few of these alternative materials exhibit overall HER performance (such as activity and durability) that matches that of commercial Pt/C catalysts. In addition, the vast majority of these strategies for reducing or replacing Pt involve specialized equipment, complicated and time‐consuming synthetic processes and/or expensive reagents, making them impractical for large‐scale applications.^1^, ^2^, ^3^ Therefore, developing practical methods for the synthesis of HER catalysts with superior performance and ultra‐low Pt loadings remains an important goal.

Recent HER trials have shown that Pt counter‐electrodes can slowly dissolve in H_2_SO_4_ solutions, after which the Pt is redeposited on the surface of the working electrode.^24^, ^25^ This interesting discovery suggests the possibility of developing ultra‐low Pt catalysts with excellent HER performance, however, certain crucial issues must still be resolved. First, because multiple reactions, including the re‐deposition of Pt, the HER and the reactions of the catalysts themselves, occur simultaneously only at the work electrode during the HER test, it is necessary to determine the manner in which these complex reactions affect the catalyst formation mechanism. Second, it is necessary to investigate improvement of the overall HER performance while reducing the Pt loading of hybrid catalysts. Last, it would be beneficial to determine if the use of various metals other than Pt as the counter‐electrode can also improve the HER performance. The purpose of the present work was to explore these topics. To the best of our knowledge, although some Pt hybrid catalysts have been synthesized using Pt counter‐electrode, such as Pt‐WS_2_‐nanosheet structures^26^ and single Pt/CoP‐based nanotubes,^22^ systematic studies involving these crucial issues have rarely been reported.

In the present study, we applied the sacrificial‐counter‐electrode method for the synthesis of MoS*_x_*/carbon nanotubes (CNTs)/Pt catalysts (MoS*_x_*/CNTs/Pt). This work determined that a portion of the S atoms in MoS*_x_* can be substituted with O atoms during a potential cycling process when using Pt as a counter‐electrode. This process generates a MoS*_x_*–O–PtO*_x_* structure in the MoS*_x_*/CNTs/Pt hybrid catalyst. Density functional theory (DFT) calculations indicate that the O atoms in the MoS*_x_*–O–PtO*_x_* structure act as bridges between the catalytic PtO*_x_* particles and the MoS_2_ support, allowing the Pt atoms to donate more electrons and thus facilitating the HER process. The experimental results also confirm that this MoS*_x_*/CNTs/Pt hybrid catalyst (0.55 wt% Pt loading) exhibits a low overpotential of 25 mV at a current density of 10 mA cm^−2^, a low Tafel slope of 27 mV dec^−1^, and excellent stability under acidic conditions during the HER. The overall HER performance of this material is superior to that of current commercial Pt/C catalyst (20 wt% Pt loading). More importantly, it was found that other non‐Pt metals (such as Pd and W) could be employed as the sacrificial counter‐electrode and that these also improved the HER performance of the MoS*_x_* catalyst, further increasing the feasibility of using this technique for practical applications in the future.

## 2 Results and Discussion

**Figure 1:**
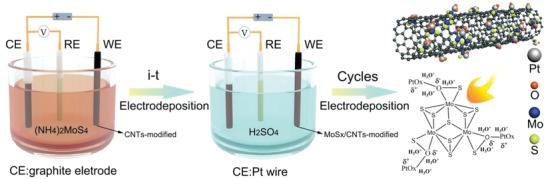
Schematic illustration of the MoS*_x_*/CNTs/Pt synthesis procedure.

**Figure** **1** summarizes the entire synthetic procedure used to obtain the MoS*_x_*/CNTs/Pt catalyst. Briefly, the hybrid catalysts were synthesized via a two‐step electrochemical process (see Supporting Information for experimental details). Initially, a CNT‐modified glass carbon electrode (GCE) was soaked in an aqueous solution of (NH_4_)_2_MoS_4_ and NaClO_4_, such that MoS*_x_* was deposited on the surfaces of the CNTs via an amperometric *i*–*t* curve (*i*–*t*) process. The samples obtained after various reaction times are denoted herein as shown in Table S1 in the Supporting Information. Figure S1 and Table S1 in the Supporting Information demonstrate that the MoS*_x_*/CNTs sample produced using a reaction time of 1800 s generated a higher catalytic current than the other materials, and so this specimen was employed in the subsequent experimentation. The MoS*_x_*/CNTs‐modified GCEs were soaked in aqueous H_2_SO_4_ solutions and the potential cycling was carried out using a Pt wire as the sacrificial counter‐electrode in a three‐electrode system. Parallel experimental trials employing various numbers of potential cycles were performed (Figure S2, Supporting Information) and the resulting catalysts are denoted herein as MoS*_x_*/CNTs/Pt_5k_, MoS*_x_*/CNTs/Pt_10_ *_k_*, and so on, where the sign of “*k*” indicates the cycles number of 1000. Their HER performances were evaluated using a graphite rod as a counter‐electrode in a standard three‐electrode system. From the data in Table S1 in the Supporting Information, it is evident that the MoS*_x_*/CNTs/Pt_10_ *_k_* sample produced the highest catalytic current, and so this sample was selected as the optimal host material for subsequent experiments.

**Figure 2:**
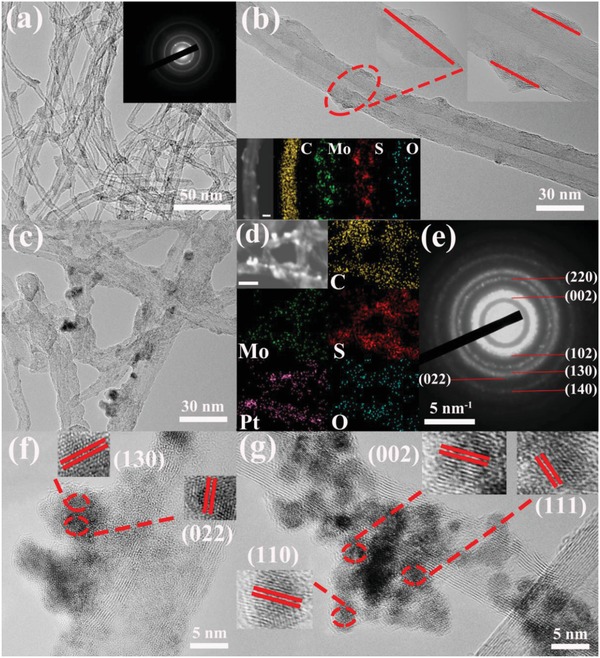
Characterizations of the MoS*_x_*/CNTs hybrid catalysts: a,b) TEM images of a MoS*_x_*/CNTs hybrid catalyst. The inset to (a) is the electron diffraction pattern and the insets to (b) in the lower left and upper right show the EDX elemental maps for C, Mo, S, and O (scale bar = 200 nm) and an HRTEM image. Characterizations of the MoS*_x_*/CNTs/Pt_10_ *_k_* hybrid catalysts: c) TEM image, d) STEM and corresponding element maps (scale bar = 500 nm), e) electron diffraction pattern, f,g) HAADF‐STEM and corresponding HRTEM images.

Transmission electron microscopy (TEM) images of the MoS*_x_*/CNTs are presented in **Figure** **2**a. These images demonstrate that particles were dispersed on the CNT surfaces, while the high‐resolution TEM (HRTEM) image in Figure 2b also shows a thin amorphous coating over the CNTs. Energy‐dispersive X‐ray (EDX) mapping confirmed that both Mo and S were uniformly distributed over the CNTs (Figure 2b, lower left), suggesting that MoS*_x_* was present on the CNTs. The selected‐area electron‐diffraction (SAED) characterization in the inset to Figure 2a contains blurry rings generated by the MoS*_x_*/CNTs that imply nonperfect ordering of the MoS*_x_*, further confirming that thin layers of amorphous MoS*_x_* were successfully applied to the CNTs.^27^ Figure 2c and Figure S3 in the Supporting Information show SEM and TEM images of the MoS*_x_*/CNTs/Pt_10_ *_k_*. In contrast to the MoS*_x_*/CNTs TEM image in Figure 2b, the HRTEM images in Figure 2f,g show two phases very clearly, one is amorphous and the other consists of distinct lattice fringes. The fringe spacings of 0.35 and 0.29 nm represent the (130) and (022) planes of the PtO_2_, while the HRTEM image in Figure 2g indicates the (002), (111), and (110) crystal grains of PtO. These results are also consistent with the SAED data in Figure 2e, which exhibit several bright rings attributed to PtO*_x_* crystal grains. These observations together with the EDX mapping results in Figure 2d confirm that Pt was uniformly distributed over the MoS*_x_*/CNTs/Pt_10_ *_k_* via the potential cycling process. An inductively coupled plasma optical emission spectroscopy (ICP‐OES) analysis determined that the MoS*_x_*/CNTs/Pt_10_ *_k_* samples had a 0.55 wt% Pt loading.

**Figure 3:**
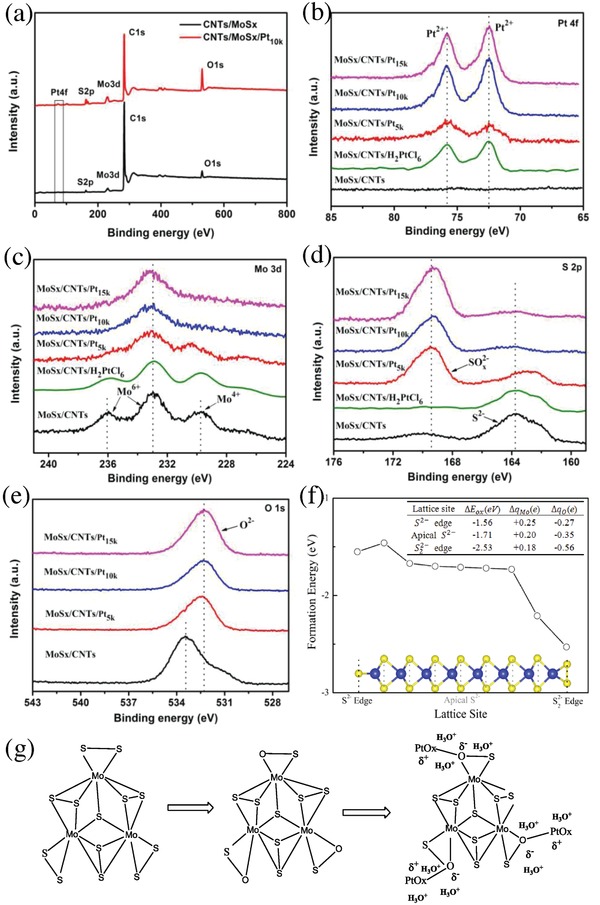
Structural characterizations of the catalyst: a) the XPS patterns of the MoS*_x_*/CNTs (black line) and MoS*_x_*/CNTs/Pt_10_ *_k_* (red line). The b) Pt 4f, c) Mo 3d, d) S 2p, and e) O 1s spectra of the hybrid catalysts. f) The calculated formation energy profiles for the oxidation of a single 2H–MoS_2_ sheet at different S lattice sites. The inset presents a front view of a MoS_2_ nanosheet, showing terminal $S_{2}^{2-}$ or S^2−^ sites on the edge and apical S^2−^ sites on the basal surface. The yellow and blue spheres indicate S and Mo atoms. The table summarizes the Bader charge analysis of the three major oxidation scenarios. g) A schematic illustrating the change in catalyst structure during the electrodeposition process.

The chemical compositions of the pristine MoS*_x_*/CNTs and the MoS*_x_*/CNTs/Pt products made with various numbers of potential cycles were assessed by X‐ray photoelectron spectroscopy (XPS). **Figure** **3**a confirms the presence of C, Mo, S, O, and Pt in the MoS*_x_*/CNTs/Pt_10_ *_k_*, while Figure 3b shows the Pt 4f spectrum in which there are peaks at 72.5 and 75.8 eV that are characteristic of Pt^2+^. Changes in the chemical states of the elements in the MoS*_x_*/CNTs/Pt samples following the application of potential cycles were also compared. From Figure 3c,d, it is apparent that the Mo 3d and S 2p peaks were shifted to higher binding energies (BEs) after the potential cycling process, demonstrating that the Mo(IV) (at 229.6 eV) transitioned to Mo(VI) (at 233 eV) and the S^2−^ (at 163.6 eV) gradually transformed to SO*_x_* ^2−^ (at 169.2 eV).^28^ It is also apparent that the O 1s spectra of the MoS*_x_*/CNTs/Pt samples in Figure 3e show a shift toward lower BEs compared with the MoS*_x_*/CNT sample. The peak at 532.2 eV is assigned to divalent oxygen (O^2−^), which indicates that more O^2−^ appears in these MoS*_x_*/CNTs/Pt samples. The aforementioned XPS results also suggest that the MoS*_x_* was susceptible to oxidation. That is, some of the S atoms in the MoS*_x_* would have been replaced by O atoms during the potential cycling process when using Pt as a sacrificial counter‐electrode, after which these O atoms would serve as bridges between the PtO*_x_* and MoS*_x_*. To gain further insights into the correlation between the O substitution and the potential cycling process using Pt as the sacrificial counter‐electrode. We also designed a comparative experiment using the prepared MoS*_x_*/CNTs material as the working electrode to electrodeposit Pt directly in chloroplatinic acid (H_2_PtCl_6_) solution (see Supporting Information for experimental details). The samples obtained using various numbers of electrodeposition cycles are denoted herein as MoS*_x_*/CNTs/H_2_PtCl_6_^1^, MoS*_x_*/CNTs/H_2_PtCl_6_, and MoS*_x_*/CNTs/H_2_PtCl_6_^2^ (Figures S4 and S5, Supporting Information). The MoS*_x_*/CNTs/H_2_PtCl_6_ sample with 0.54 wt% Pt loading was also analyzed by XPS and the Mo 3d and S 2p spectra in Figure 3c,d demonstrate no signs of O substitution. These observations further confirm that the reaction involving S substitution by O primarily occurred during the potential cycling process when using Pt wire as the sacrificial counter‐electrode.

Assuming that the substitution of S atoms by O (that is, the formation of O_S_ defects) was thermodynamically competitive with O adsorption,^29^ we investigated the atomic‐scale mechanism of MoS_2_ oxidation based on theoretical calculations. Figure 3f presents the formation energy profile for O substitution of a single sheet of 2H–MoS_2_ at different S sites. The DFT calculations yielded negative values for the oxidation, demonstrating a thermodynamically stable substitution process. The formation energy for the replacement of a single S^2−^ edge atom by O was found to be approximately −1.56 eV, while the value for replacement of an apical S^2−^ site (in the central region of the MoS_2_ nanosheet) was −1.71 eV. It is worth noting that O substitution at the $S_{2}^{2-}$ edge sites yielded the energy of oxidation was −2.53 eV, which is significantly different from the others, and suggests that the $S_{2}^{2-}$ edges were most likely to be substituted by atomic O. We also conducted a Bader charge analysis of the three major oxidation sites within the nanostrip, with the results in the tabular inset to Figure 3f. For a single S (S^2−^) edge, the charge difference for O_S_ was approximately −0.27 *e*, which was primarily transferred from two neighboring Mo atoms (+0.25 *e* from both). With regard to the apical S^2−^ sites, the value was approximately −0.35 *e* more than the substituted S positions, with three Mo neighbors donating +0.20 *e* each due to the relatively greater electronegativity of O. Most importantly, the largest charge deviation associated with oxidation was at the $S_{2}^{2-}$ edge, with the minimum amount of electron transfer from Mo atoms. These results provide further evidence that the $S_{2}^{2-}$ edge could be highly susceptible to oxidation. According to the experimental data, we devised a mechanism for the formation of MoS*_x_*/CNTs/Pt via potential cycling, as shown in Figure 3g.

**Figure 4:**
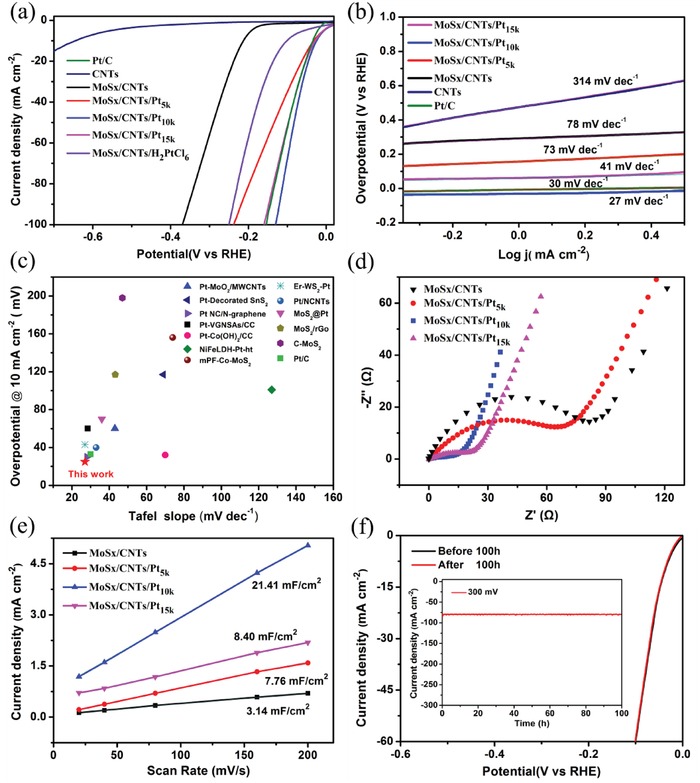
The HER performance of the catalysts: a) the polarization curves for CNTs, Pt/C, MoS*_x_*/CNTs and MoS*_x_*/CNTs/Pt in a 0.5 m H_2_SO_4_ solution at a scan rate of 10 mV s^−1^ and b) the corresponding Tafel plots. c) A comparison of the overpotentials at a current density of 10 mA cm^−2^ and the Tafel slopes for the various catalysts. d) The Nyquist plots for the MoS*_x_*/CNTs/Pt hybrid catalysts. e) The double‐layer capacitance values for the MoS*_x_*/CNTs/Pt hybrid catalysts. f) Linear sweep voltammetry curves for MoS*_x_*/CNTs/Pt_10_ *_k_* before and after 100 h. The inset shows the chronoamperometry *i*–*t* curve for MoS*_x_*/CNTs/Pt_10_ *_k_* at η = 300 mV in 0.5 m H_2_SO_4_.

The HER activities of the various MoS*_x_*/CNTs/Pt catalysts were evaluated under acidic conditions (0.5 m H_2_SO_4_) using a graphite rod as a counter‐electrode in a three‐electrode configuration. The results were compared with those obtained from pristine CNTs, a commercial Pt/C catalyst, and the MoS*_x_*/CNTs/H_2_PtCl_6_. The HER polarization curves of the MoS*_x_*/CNTs catalysts after 5000, 10 000, and 15 000 potential cycles are presented in **Figure** **4**a. The *ŋ* values at *j*_HER_ = 10 mA cm^−2^ are widely used to evaluate the HER electrocatalytic performance, with smaller values indicating higher HER activities. The MoS*_x_*/CNTs/Pt_10_ *_k_* catalyst demonstrated significant activity during the HER, which achieves the current density of 10 mA cm^−2^ only need the 25 mV overpotential. The Tafel slopes based on polarization curves are presented in Figure 4b. The Tafel slope for the MoS*_x_*/CNTs/Pt_10_ *_k_* was 27 mV decade^−1^, which is equal to that for the commercial Pt/C (30 mV decade^−1^) in an acidic solution. These results demonstrate that the HER on both the MoS*_x_*/CNTs/Pt_10_ *_k_* and Pt/C proceeded via the Volmer–Tafel mechanism, with recombination between adjacent adsorbed H atoms as the rate‐limiting step (RLS).^30^, ^31^ Figure 4b presents the Tafel slope for the CNTs, MoS*_x_*/CNTs, MoS*_x_*/CNTs/Pt_5_ *_k_*, MoS*_x_*/CNTs/Pt_15_ *_k_* and other specimens, which may proceed through the Volmer–Heyrovsky mechanism. This involves the electrochemical combination of an adsorbed H atom, a free proton and a free electron as the RLS.^30^, ^31^ To better compare the HER kinetics to the recently reported results for a highly active low‐Pt catalyst in an acidic electrolyte, the overpotential at a current density of 10 mA cm^−2^ and the Tafel slope are summarized in Figure 4c. The HER performance of our MoS*_x_*/CNTs/Pt_10_ *_k_* hybrid catalyst is better than those of low‐Pt catalysts and is also comparable to that of any HER catalyst reported to date (Table S3, Supporting Information).

The origin of the high HER activity exhibited by the MoS*_x_*/CNTs/Pt_10_ *_k_* was examined by performing an electrochemical impedance spectroscopy (EIS) analysis of the MoS*_x_*/CNTs/Pt catalysts. The resulting Nyquist plots are shown in Figure 4d, and the electrical equivalent circuit diagram in Figure S6 in the Supporting Information was used to model the solid–liquid interface after the experimental data were well fitted (Figure S7, Supporting Information). The impedance parameters obtained by fitting the EIS responses are summarized in Table S2 in the Supporting Information. These data demonstrate that the MoS*_x_*/CNTs/Pt_10_ *_k_* had a low Rp value of 52 Ω and the largest electric double layer capacitance (CPE) value among all of the composite catalysts. This low Rp value indicates rapid surface charge transfer and a higher reaction rate in the electrocatalysis kinetics.^9^ The large CPE value corresponds to a high active surface area, which would be expected to greatly promote the HER performance of the material. These may be the main reasons why the MoS*_x_*/CNTs/Pt_10_ *_k_* had the highest HER activity among all the composite catalysts. The electrochemical surface area values were also evaluated to gain further insights into these electrocatalysts. These values were obtained by calculating the electrochemical double layer capacitances (*C*_dl_) utilizing a simple cyclic voltammetry method, and the standard CV curves acquired for the different materials with varying scan rates are shown in Figure S8 in the Supporting Information. Here, a larger *C*_dl_ value implies a higher effective active surface area. As shown in Figure 4e, the *C*_dl_ of the MoS*_x_*/CNTs/Pt_10_ *_k_* was approximately 21.41 mF cm^−2^ and thus much higher than the values for the MoS*_x_*/CNTs, MoS*_x_*/CNTs/Pt_5_ *_k_*, and MoS*_x_*/CNTs/Pt_15_ *_k_*. The SEM images of these MoS*_x_*/CNTs/Pt samples are shown in Figure S9 in the Supporting Information and a morphology analysis suggests that the porosity of these materials actually increased along with the number of potential cycles. This result may explain why the MoS*_x_*/CNTs/Pt composites made with fewer deposition cycles (fewer than 10 000 cycles) had fewer active sites. Moreover, the ICP‐OES analysis demonstrates that the Pt concentration in the MoS*_x_*/CNTs/Pt_15_ *_k_* was 0.53 wt%, and so slightly lower than that in the MoS*_x_*/CNTs/Pt_10_ *_k_*. This can be explained by the shedding of Pt during longer potential cycling (greater than 10 000 cycles), which was also confirmed by our visual observations.

The stability of the catalyst is also an important factor with regard to the HER, and the stability of the MoS*_x_*/CNTs/Pt_10_ *_k_* electrode was evaluated by monitoring the current density during continuous operation at 0.3 V (vs a reversible hydrogen electrode (RHE)) under acidic conditions. The corresponding time‐dependent current density curves are shown in Figure 4f, and these data confirm that the current density of the catalyst was constant for over 100 h. The polarization curve of the MoS*_x_*/CNTs/Pt_10_ *_k_* catalyst after 100 h is almost identical to the initial one (Figure 4f), suggesting negligible loss of cathodic current. This in turn indicates that the MoS*_x_*/CNTs/Pt_10_ *_k_* catalyst exhibits excellent durability under acidic conditions. This level of durability is much greater than those of the majority of previously reported Pt hybrid catalysts (Table S3, Supporting Information). As demonstrated by the aforementioned XPS analysis (Figure 3), the strong interactions between the PtO*_x_* and MoS*_x_*, rooted in the bridging effect of the substituted O atoms, are evidently an important factor in achieving this remarkable level of robustness.

**Figure 5:**
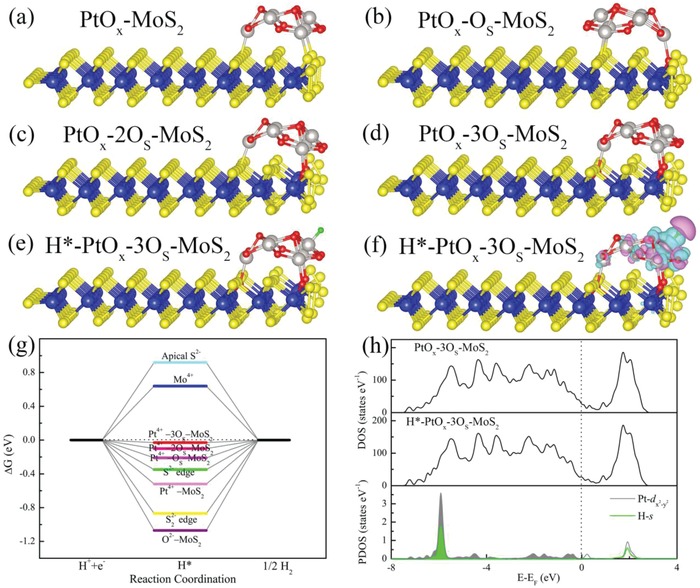
Schematic front views of the optimal 3D geometries of PtO*_x_* clusters deposited on MoS_2_ nanosheets with varying levels of oxidation: a) the bare $S_{2}^{2-}$ edge, b) one O_S_ defect, c) two O_S_ defects, and d) three O_S_ defects. The yellow, red, white, and blue spheres indicate S, O, Pt, and Mo atoms. e) The optimal structure model of a PtO*_X_* cluster generated on an oxidized MoS_2_ basal plane with H* absorption at a Pt^4+^ site. f) Differences in the charge density around the H*—Pt bond. The charge is localized primarily between the bond (the light green region) and is less concentrated outside this region (the purple region), with a value of 0.02 electrons Å^−3^. g) An HER free‐energy diagram for different H* absorption sites on the complex catalyst. h) Density of states for the PtO*_x_*–3O_S_–MoS_2_ and H*–PtO*_x_*–3O_S_–MoS_2_ and PDOSs for the Pt‐d*_x2‐y2_* and H‐s of H*–PtO*_x_*–3O_S_–MoS_2_.

Figure 4a also shows that the MoS*_x_*/CNTs/H_2_PtCl_6_ catalyst required a higher overpotential to achieve a 10 mA cm^−2^ currently density compared with that for the MoS*_x_*/CNTs/Pt_10k_. Based on structural characterizations, we can safely conclude that O substitution played a key role in the excellent HER performance of this material. To gain further insights into the effect of O substitution on the location of PtO*_x_* clusters, we examined Pt_6_O_9_ via quantum mechanical DFT calculations,^32^, ^33^ and found that each Pt atom in the cluster was coordinated with four O atoms with a bond length of approximately 2.04 Å. The optimal geometries for Pt_6_O_9_ adsorption on an oxidized MoS_2_ nanosheet were determined and are shown in **Figure** **5**. According to the optimized cluster structure, each Pt atom was coordinated with four O atoms and the Pt—O bond length was approximately 2.04 Å. The four coordinated O atoms around the central Pt ion formed a square planar structure with D 4h symmetry. The adsorption energies indicate that PtO*_x_* clusters will be preferentially deposited on the $S_{2}^{2-}$ edge sites rather than other regions of the MoS_2_ nanostrips, in agreement with previous reports.^26^ Figure 5a displays the 3D structure of Pt_6_O_9_ generated on the bare $S_{2}^{2-}$ edge of MoS_2_, while Figure 5b–d shows the geometric models after optimization, in which a Pt_6_O_9_ cluster is deposited on the disulfide terminal edge sites of MoS_2_ nanosheets with varying levels of oxidation. The formation energies for Pt_6_O_9_ on the terminal $S_{2}^{2-}$ edge were calculated to be −6.02 eV with one O_S_ defect, −6.35 eV with two O_S_ defects, and −6.74 eV with three O_S_ defects, all of which are much lower than the value for Pt_6_O_9_ located on the bare $S_{2}^{2-}$ edge (−5.17 eV) on the MoS_2_ surface. Therefore, the O_S_ antisite defects undergo a much stronger interaction with the PtO*_x_* clusters compared with the bare $S_{2}^{2-}$ edge, suggesting that substituted O atoms strengthen the bridging between the catalytic PtO*_x_* particles and MoS_2_ support. These results also indicate that this catalyst, having a MoS*_x_*–O–PtO*_x_* structure, has potential applications in electrocatalytic hydrogen evolution.

To further elucidate the catalytic mechanism during the HER on the MoS*_x_*/CNTs/Pt catalytic system, a Pt_6_O_9_ nanoparticle was constructed on a 2H–MoS_2_ basal surface. After completing the structural optimization (as shown in Figure 5e), the Pt^4+^ was coordinated with one H*. The bond length was found to be approximately 1.53 Å, which is close to the length obtained from the experimental results. The charge density differences between the states before and after hydrogen bonding are presented in Figure 5f. Note that the charge is localized between the Pt—H bond (the light green zone) and is less concentrated outside this region (the purple zone), which is typical of an s‐type valence bond. The projected density of states (PDOSs) for the Pt d*_xy_*, Pt d*_x2‐y2_*, and H 1s orbitals of the H*–PtO*_x_*/MoS_2_ are all presented in Figure 5h, and confirm that the H 1s state is primarily hybridized with the Pt d*_x2‐y2_* orbital rather than the others. In the case of an HER catalyst, the relative free energy of H* absorption on the surface is the key to evaluating catalytic activity. As shown in Figure 5g, the calculated ∆*G* value for H* adsorption on the O^2−^ of a PtO*_x_* cluster is on the order of −1.09 eV, which is consistent with prior reports. The absolute ∆G value for the HER on Pt^4+^ gradually approaches zero (−0.03 eV for the Pt^4+^ ions of PtO_x_‐3O_S_‐MoS_2_) with increases in the extent of oxidation of the MoS_2_. In this work, we found that the free energy for H* adsorption on Pt^4+^ changes to +0.16 eV when all the O^2−^ ions in the PtO_x_ cluster are initially passivated by H. Moreover, the relative energies for the sorption of H at apical S^2−^ and Mo^4+^ sites have large positive values, while the S^2−^ and $S_{2}^{2-}$ edge sites show the negative ones on the MoS_2_ basal surface. Therefore, we conclude that the Pt^4+^ ions in the PtO_x_‐3O_S_‐MoS_2_ are the most active catalytic sites during the HER. As shown in Figure 5h, the partially occupied surface state was situated at the Fermi level of the PtO*_x_*–3O_S_–MoS_2_. The adsorption of H* on the PtO*_x_* cluster generated an H 1s state that was primarily in the vicinity of −5.8 eV, and the Pt‐d*_x2‐y2_* and H‐s orbitals were able to form a weak valence bond. Although the Bader charge analysis shows no obvious charge transfer between the PtO*_x_* cluster and the H, the charge density difference analysis in Figure 5f clearly demonstrates charge transfer from H* to a Pt atom of the PtO*_x_* cluster, explaining the high catalytic activity of the PtO*_x_*–3O_S_–MoS_2_ during the HER process.

**Figure 6:**
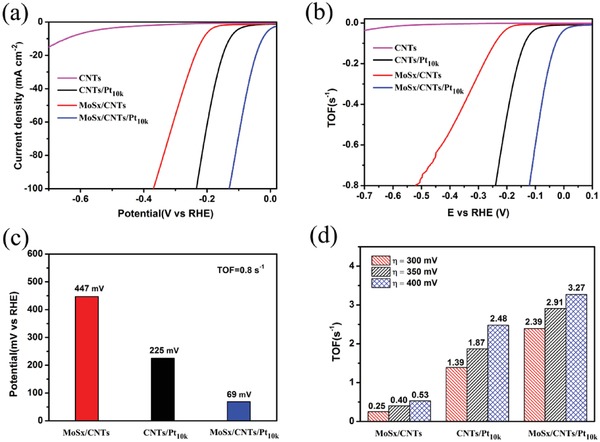
The TOF values of the catalysts: a) The polarization curves for CNTs, CNTs/Pt_10_ *_k_*, MoS*_x_*/CNTs, and MoS*_x_*/CNTs/Pt_10_ *_x_* in a 0.5 m H_2_SO_4_ solution acquired at a scan rate of 10 mV s^−1^. b) The calculated TOF values for CNTs, CNTs/Pt_10_ *_k_*, MoS*_x_*/CNTs, and MoS*_x_*/CNTs/Pt_10_ *_x_* from experimental data. c) A comparison of the overpotentials of samples at the same TOF values. d) A comparison of the TOF values of samples at the same overpotentials.

The turnover frequency (TOF) is a useful parameter for assessing the synergistic enhancement effects of O substitution. An experiment was therefore designed to allow direct electrodeposition of the same amount of Pt on a CNTs‐modified GCE, and the resulting sample is denoted CNTs/Pt_10_ *_k_*. The number of active sites in this material was estimated by using cyclic voltammetry to examine the different catalyst electrodes in 1.0 m phosphate buffered saline (pH = 7) over the potential window from −0.2 to 0.6 V versus RHE at a scan rate of 50 mV s^−1^ (Figure S10, Supporting Information, for details of the calculation method and experimental process).^34^, ^35^ The HER polarization curves for the different catalysts are provided in **Figure** **6**a and clearly show that the HER activities of the CNTs/Pt_10_ *_k_* and MoS*_x_*/CNTs were lower than that of the MoS*_x_*/CNTs/Pt_10_ *_k_*. Figure 6b shows the different TOF curves for the CNTs, CNTs/Pt_10_ *_k_*, MoS*_x_*/CNTs, and MoS*_x_*/CNTs/Pt_10_ *_k_* in 0.5 m H_2_SO_4_ within the range of applied potentials. Approximate TOF values can be used to compare the activity of the MoS*_x_*/CNTs/Pt_10_ *_k_* with those of the other catalysts. Figure 6c demonstrates that overpotentials of 447 and 225 mV were required to achieve the same TOF of 0.8 s^−1^ for the MoS*_x_*/CNTs and CNTs/Pt_10_ *_k_*, while the MoS*_x_*/CNTs/Pt_10_ *_k_* only required 69 mV to reach the same value. This value is much lower than those for the MoS*_x_*/CNTs and CNTs/Pt_10_ *_k_*. To make a clear comparison, we also compared the TOF values at overpotentials of 300, 350, and 400 mV. As shown in Figure 6d, the TOF values for the MoS*_x_*/CNTs/Pt_10_ *_k_* were always greater than the combined values for the MoS*_x_*/CNTs and CNTs/Pt_10_ *_k_*. This result demonstrates the synergistic enhancement effects of O substitution on the catalyst with the MoS*_x_*–O–PtO*_x_* structure. Then the Faradic efficiency (FE) of MoSx/CNTs/Pt_10_ *_k_* for water electrolysis was further evaluated by gas chromatography. The amount of H_2_ increases during electrolysis process and the approximate agreement of measured value and theoretical one suggests nearly 100% FE (Figure S11, Supporting Information, for details of the calculation method and experimental process).^36^

The above findings raised the intriguing possibility of the general applicability of this method involving the sacrifice of a counter‐electrode. Therefore, we also selected some inexpensive wires made of metals other than Pt, such as Pd wire and W filament, for use as sacrificial counter‐electrodes and carried out similar experiments (see Supporting Information for experimental details). The HER polarization curves for the MoS*_x_*/CNTs, MoS*_x_*/CNTs/W_10_ *_k_*, and MoS*_x_*/CNTs/Pd_2_ *_k_* and the corresponding Tafel slopes are shown in Figure S12 in the Supporting Information. It can be seen that using either Pd or W wire as the counter‐electrode produced an obvious improvement in the HER performance of the MoS_x_/CNT sample. Specifically, the MoS*_x_*/CNTs/Pd_2_ *_k_* catalyst also exhibited excellent HER performance comparable to that of the MoS*_x_*/CNTs/Pt_10_ *_k_* catalyst. These encouraging results provide further evidence that this method involving the sacrifice of a counter‐electrode could be a promising route to the synthesis of catalysts with remarkable HER performance.

## 3 Conclusions

In summary, we have demonstrated a novel strategy involving the sacrifice of a counter‐electrode to successfully synthesize a MoS*_x_*/CNTs/Pt_10_ *_k_* catalyst with an ultra‐low Pt loading of 0.55 wt%. This material exhibits excellent HER performance, with a small overpotential of 25 mV at a current density of 10 mA cm^−2^ and a low Tafel slope of 27 mV dec^−1^. The catalyst also shows good catalytic stability over at least 100 h with almost no current attenuation in acidic media, such that the HER activity of the MoS*_x_*/CNTs/Pt_10_ *_k_* is even better than that of Pt/C in acidic solutions. DFT calculations indicate that substituted O atoms strengthen the bridging between catalytic PtO*_x_* particles and MoS_2_ supports, such that the Pt atoms donate more electrons, which facilitates the HER process. This synthesis method involving a sacrificial counter‐electrode may offer a new approach to developing various hybrid materials with ultra‐low transition metal loadings for applications in electrocatalytic fields.

## Conflict of Interest

The authors declare no conflict of interest.

## Supporting information

Supplementary
