# Supplementary material for: Synthesis of a MoSx–O–PtOx Electrocatalyst with High Hydrogen Evolution Activity Using a Sacrificial Counter‐Electrode
Source: Adv Sci (Weinh). 2019 Jan 12;6(5):1801663. doi: 10.1002/advs.201801663 (PMC6402408; doi:10.1002/advs.201801663)
Supplement: Supplementary file 1 — Supplementary [file ADVS-6-1801663-s001.pdf]

Copyright WILEY-VCH Verlag GmbH & Co. KGaA, 69469 Weinheim, Germany, 2019.

ADVANCED SCIENCE
Open Access

Supporting Information

for *Adv. Sci.,* DOI: 10.1002/advs.201801663

Synthesis of a MoS_x–O–PtO_x Electrocatalyst with High Hydrogen Evolution Activity Using a Sacrificial Counter-Electrode

Yingxin Zhan, Yi Li, Zhi Yang, Xiongwei Wu, Mengzhan Ge, Xuemei Zhou, Junjie Hou, Xiannuo Zheng, Yuchong Lai, Rongrong Pang, Huan Duan, Xi'an Chen, Huagui Nie,* and Shaoming Huang**

Synthesis of a MoS_x-O-PtO_x Electrocatalyst with High Hydrogen Evolution Activity using a Sacrificial Counter Electrode

Yingxin Zhan, Yi Li, Zhi Yang*, Xiongwei Wu, Mengzhan Ge, Xuemei Zhou, Junjie Hou, Xiannuo Zheng, Yuchong Lai, Rongrong Pang, Huan Duan, Xi'an Chen, Huagui Nie*, Shaoming Huang*

Y. Zhan, Y. Li, Prof. Z. Yang, M. Ge, X. Zhou, J. Hou, X. Zheng, Y. Lai, R. Pang, Prof. X. Chen, Prof. H. Nie, Prof. S. Huang

Nanomaterials & Chemistry Key Laboratory, Wenzhou University, Wenzhou, 325027, China

Email: yang201079@126.com, huaguinie@126.com, smhuang@wzu.edu.cn

Prof. S. Huang

School of Material and Energy, Guangdong University of Technology, Guangzhou, China

Prof. X. Wu

College of Science, Hunan Agricultural University, Changsha, Hunan 410128, China

H. Duan

School of Chemistry and Chemical Engineering, Southwest University, Chongqing, 400715, China

Experimental section

1.1 Electrode Preparation

Bare glassy carbon electrodes (GCE) (3 mm diameter, CH Instrument Inc.) were polished with different sizes alumina slurry on a microcloth and subsequently rinsed with ultrapure water and ethanol. The electrodes were then sonicated in ethanol, and dried under a gentle nitrogen stream. To prepare the working electrode, a 2 mg CNT sample (CNTs were purchased from Cnano Technology (Beijing) Limited (purity > 95%; diameter 11 nm; length = 10 μm (average); synthesis method, CVD)) was ultrasonically dispersed in the mixed solution of ethanol and H_2O (500 mL), and then 8μL of the resultant suspension was dropped onto the GCE surface and dried at room temperature. For comparison,a commercially available Pt/C-modified GCE (20 wt% Pt supported on carbon black, fuel cell grade from Alfa Aesar) was prepared in the same way.

1.2 Synthesis of MoS_x/CNTs

The hybrid catalysts were synthesized via electrochemical deposition method, wherein CNT-modified GCEs were soaked in a 2 mM $(NH_4)_2MoS_4$ aqueous solution containing 0.1M $NaClO_4$, and the MoS_x was deposited in situ onto CNTs by i-t experiment. At the end of deposition, the working electrode was rinsed with water gently and dried at room temperature overnight. All of the potentials in our paper are calibrated to a reversible hydrogen electrode (RHE) based on the Nernst equation. For comparison, the parallel experiments using various deposition times were also carried out.

1.3 Synthesis of MoS_x/CNTs/Pt

The MoS_x/CNTs catalyst electrode was used as working electrode, a Pt wire as counter electrode, and a SCE (3 M KCl filled) electrode as reference in 0.5 M H_2SO_4 solutions, and electrodeposited in the potential range from -0.2 to -0.7V for cyclic voltammograms (CV) at a scan rate of 100 mV/s. At the end of deposition, the working electrode was rinsed with water gently and dried at room temperature overnight. The parallel experiments using various deposition cycles were also carried out. For comparison, we also use MoS_x/CNTs catalyst electrode as working electrode and the graphite rod as counter electrode, and CV in 0.025 mM $H_2PtCl_6 \cdot 6H_2O$, the everything else is the same.

1.4 Synthesis of MoS_x/CNTs/W_{10k} and MoS_x/CNTs/Pd_{2k}

The MoS_x/CNTs catalyst electrode was used as working electrode, a W or Pd wire as counter electrode, and a SCE (3 M KCl filled) electrode as reference in 0.5 M H_2SO_4 solutions, and electrodeposited in the potential range from -0.2 to -0.7V for cyclic voltammograms (CV) at a scan rate of 100 mV/s. At the end of deposition, the working electrode was rinsed with water gently and dried at room temperature overnight.

2. Structure Characterization

X-ray photoelectron spectroscopy (XPS) measurements were carried out with an ultrahigh-vacuum setup, equipped with a monochromatic Al KR X-ray source and a highresolution Gammadata-Scienta SES 2002 analyzer. SEM images were obtained with a JSM-6700 immersion scanning electron microscope. TEM analyses were

carried out with a JEM-2100F instrument operating at 200 kV. Scanning transmission electron microscopy (STEM) characterizations were performed with an aberration-corrected Titan ChemiSTEM equipped with a probe corrector (CEOS). The pt loading of samples were measured by VISTA-MPX ICP-OES.

3. Electrochemical Measurements

The electrochemical measurements were performed on a CHI 760D electrochemical workstation (Shanghai CHI Instruments Company) at 25ºC. A carbon catalyst electrode was used as working electrode, a graphite rod as the counter electrode, and a SCE (3 M KCl filled) electrode as reference. LSV in 0.5 M H_2SO_4 solutions at a scan rate of 10 mV s^{-1} to obtain the polarization curves. The long-term stability were performed by i-t tests at -0.3V (vs SCE, in 0.5 M H_2SO_4).To estimate the double-layer capacitance, cyclic voltammograms taken with various scan rates (20, 40, 80, 160, 200 mV s^{-1}) were used under the potential window of 0-0.3 V versus RHE. Electrochemical impedance spectroscopy (EIS) measurement was carried out at the open-circuit voltage with an AC voltage of 5 mV. All data were reported without iR compensation. In all measurements, we used SCE as the reference electrode. It was calibrated with respect to RHE. The calibration was performed in the high-purity hydrogen-saturated electrolyte with a Pt foil as the working and counter electrodes. Cyclic voltammetry was run at a scan rate of 1 mV s^{-1}, and the average of the two potentials at which the current crossed 0 was taken to be the thermodynamic potential for the hydrogen electrode reaction. In 0.5 M H_2SO_4, E(RHE)= E(SCE)+0.267 V.

4. TOF calculation

Cyclic voltammetry measurements of our samples were carried out in PBS electrolyte(PH=7) with a potential window from -0.2 to 0.6 V vs RHE and scan rate of 50 mV/s. Assuming one electron redox process, the integrated charge over the whole potential range was divided by two. Then, the value was divided by the Faraday constant to get the number of active sites for different samples. The turnover frequency (s^{-1}) can be estimated according to this equation:

$$TOF = I/2nF$$

where I represents the current density for different samples during the LSV measurement in 0.5 M H_2SO_4, F is the Faraday constant (C/mol), and n is the number of the active sites (mol) for different samples.

5. Computational Details

Density functional theory (DFT) calculations were performed using the all-electron, full potential electronic structure code FHI-aims at the level of the generalised gradient approximation (GGA) in the Perdew-Burke-Ernzerhof (PBE) exchange–correlation functional. Periodic boundary conditions were adopted, the structure was modelled as a nanosheet cut out from a monolayer of 2H-MoS_2, which was separated from each other by at least 12 Å in the y-direction and 20 Å in the z-direction to reduce the electrostatic interactions between them. The default "tight" basis were used in all calculations in this work. To account for the missing long-range tail of van der Waals forces, these functionals were augmented by the van der Waals scheme of Tkatchenko and Scheffler. A Gaussian occupation scheme with a smearing of 0.05 eV was used throughout.

To quantitatively access the stability of oxygen atoms at various sites of MoS_2 nanosheet, the formation energy of oxidation ($\triangle E_{ox}$) could be written as

$$\triangle E_{ox} = E_{ox} - E_{MoS_2} + \sum_i n_i u_i \qquad (1)$$

where $\triangle E_{ox}$ and E_{MoS_2} are the total energies of the oxidized system and the clean (unoxidized) MoS_2 nanosheet, n_i the number of constituent element i being added/removed from the structure, u_i is the atomic chemical potential (u_s and u_o).

According to previously published literature, the adsorption energy, which describes the stability of hydrogen adsorption, was defined by

$$\triangle E_H = E_{MoS_2+H} - E_{MoS_2} - 1/2\ E_{H_2} \qquad (2)$$

Where E_{MoS_2+H} is the total energy for one hydrogen atom adsorbed on the MoS_2 monolayer with the PtOx catalyst, E_{MoS_2} is the total energy for the MoS_2 catalytic system without hydrogen adsorption, and E_{H_2} is the total energy of a separated H_2 molecule as determined from DFT calculations.

The Gibbs free energy for atomic hydrogen adsorption was then calculated as

$$\triangle G_H = \triangle E_H + \triangle E_{ZPE} - T\triangle S_H \qquad (3)$$

Here, $\triangle E_{ZPE}$ is the zero-point energy difference between the adsorbed state of the system and the gas phase state and $\triangle S_H$ is the entropy difference between the adsorbed state of this system and the gas phase standard state (300 K, 0.1 Mpa).

The electron-density rearrangement, $\triangle \rho_{AB}$, was determined as

$$\triangle \rho_{AB} = \rho_{AB} - \rho_A - \rho_B \qquad (4)$$

From our experimental data and previous reports, we knew that the edges exposed in MoS_2 monolayer were mainly Mo edges terminated by disulfide (S_2^{2-}) or sulfur (S^{2-})

ions, and thus the diverse S species (apical S^{2-} , S^{2-} and S_2^{2-} edges) could make a tremendous impact on the oxidation and Pt nucleation owing to their different bonding characteristics. Therefore, ab initio calculations were first used to determine the energetics of atomic oxygen substitution in the MoS_2 nanosheets, followed by revealing the location of PtO_x clusters on the oxidized system and further to elucidate the triggering mechanism of this complex catalysis for HER.

6. Faradic efficiency

The Faradic efficiency is defined as the available efficiency of electrons involved in an electrochemical system. The Faradic efficiency can be calculated by the ratio of the practically produced H_2 content to its theoretical content. The former can be measured by gas chromatography. The theoretical H_2 evolution is calculated by the ratio of total buildup charge during electrolysis to the number of electrons required for H_2 evolution and Faraday's constant. The corresponding theoretically produced H_2 amount can be obtained according to this equation:

$$n_{H_2} = Q/2F$$

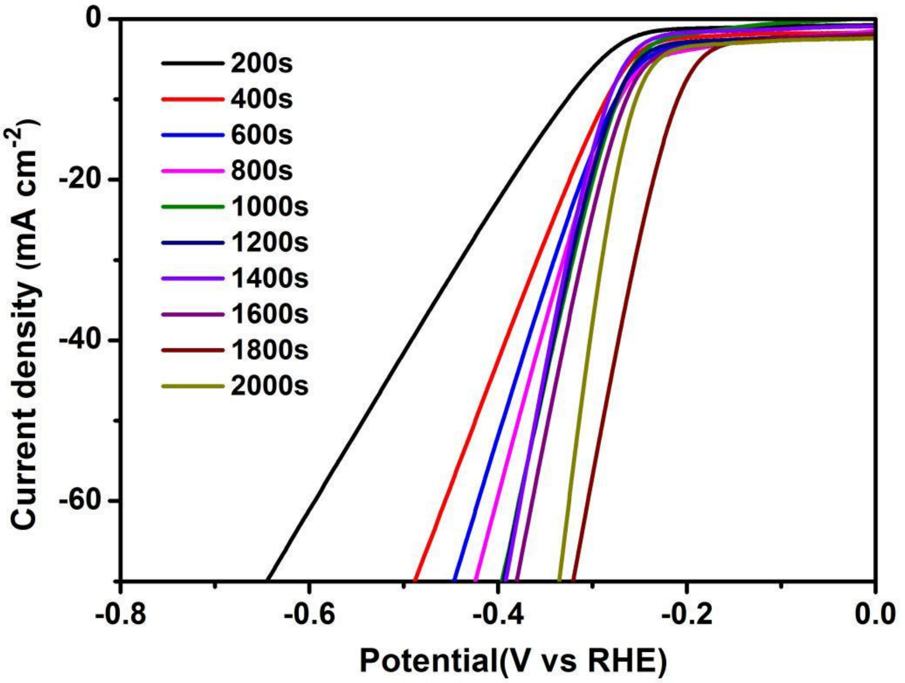

Figure S1. The polarization curves for MoSx/CNTs in various deposition times.

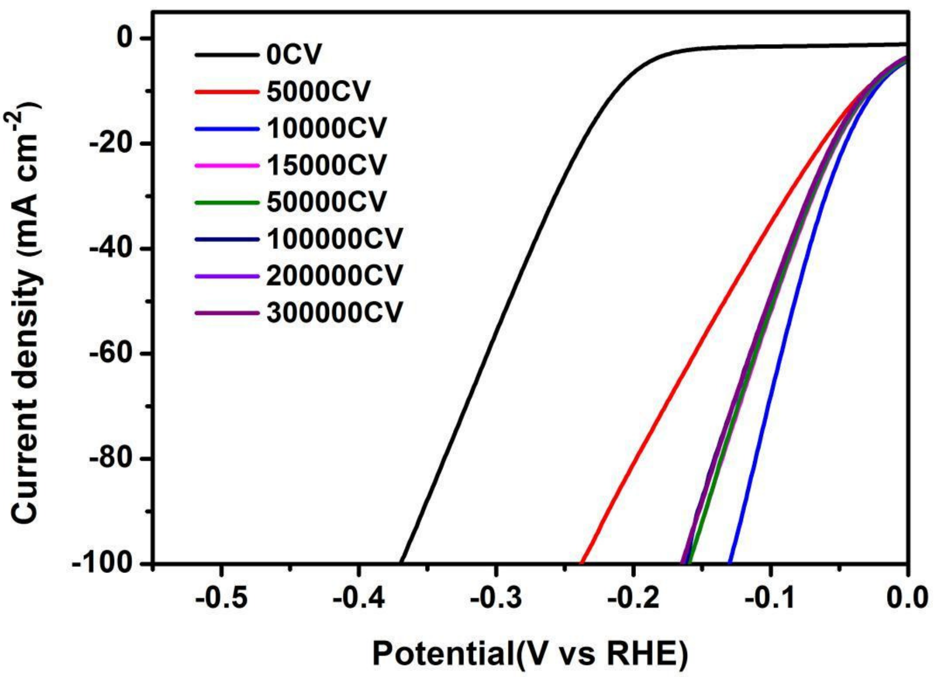

Figure S2. The polarization curves for MoSx/CNTs/Pt in different cycles

Table S1. The parallel experiments and the overpotential of different samples at a current density of 10 mA cm^{-2}

**Catalyst**	**deposition times (S)**	**deposition cycles (cycles)**	**η at corresponding $j=10mAcm^{-2}$ (mV vs RHE)**
MoSx/CNTs[1]	200		327
MoSx/CNTs[2]	400		286
MoSx/CNTs[3]	600		275
MoSx/CNTs[4]	800		273
MoSx/CNTs[5]	1000		273
MoSx/CNTs[6]	1200		274
MoSx/CNTs[7]	1400		280
MoSx/CNTs[8]	1600		265
MoSx/CNTs	1800		209
MoSx/CNTs[9]	2000		254
MoSx/CNTs/Pt_{5k}	1800	5000	32
MoSx/CNTs/Pt_{10k}	1800	10000	25
MoSx/CNTs/Pt_{15k}	1800	15000	28
MoSx/CNTs/Pt_{50k}	1800	50000	29
MoSx/CNTs/Pt_{100k}	1800	100000	31
MoSx/CNTs/Pt_{200k}	1800	200000	31
MoSx/CNTs/Pt_{300k}	1800	300000	31
Pt/C			34
MoSx/C	1800		315
pristine CNTs			645
MoSx/CNTs/H_2PtCl_6[1]	1800	50	157
MoSx/CNTs/H_2PtCl_6	1800	100	105
MoSx/CNTs/H_2PtCl_6[2]	1800	150	112
CNTs/Pt_{10k}		10000	128

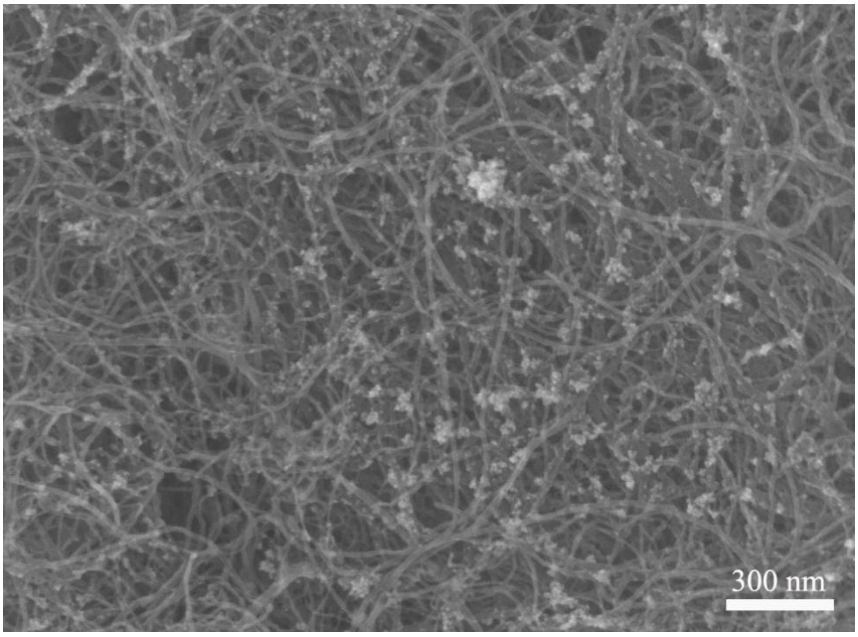

Figure S3. SEM images of MoSx/CNTs/Pt_{10k} hybrid.

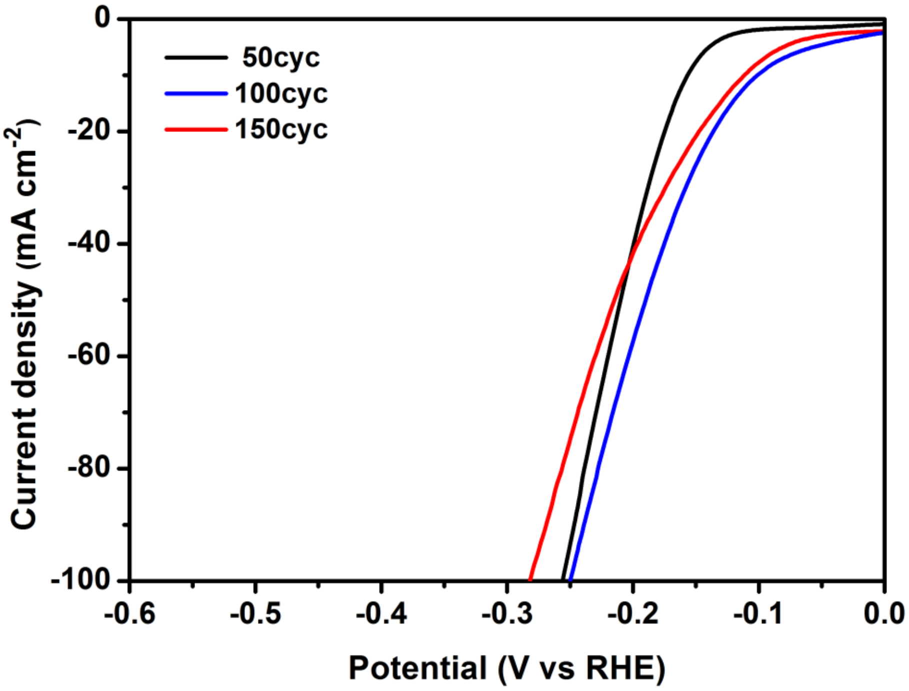

Figure S4. The HER properties with various deposition cycles of samples in chloroplatinic acid solution.

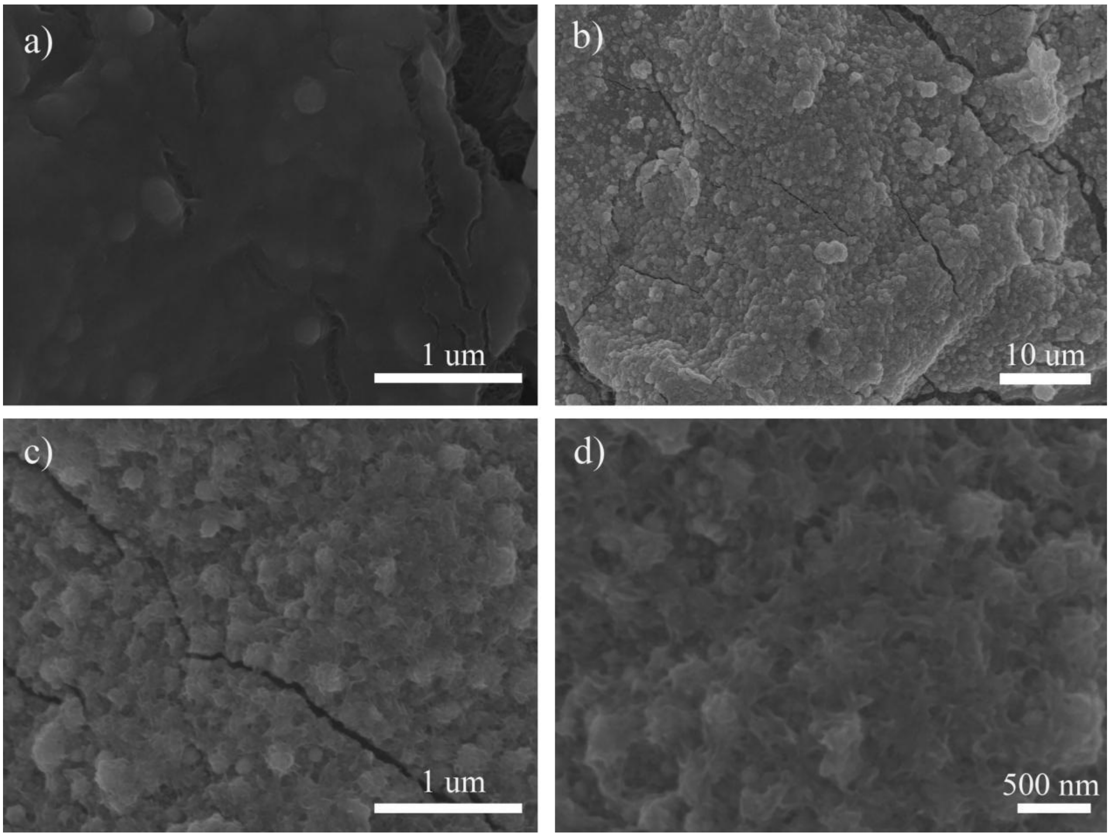

Figure S5.a,b)The SEM images of MoSx/CNTs; c,d)The SEM images of MoSx/CNTs/H_2PtCl_6.

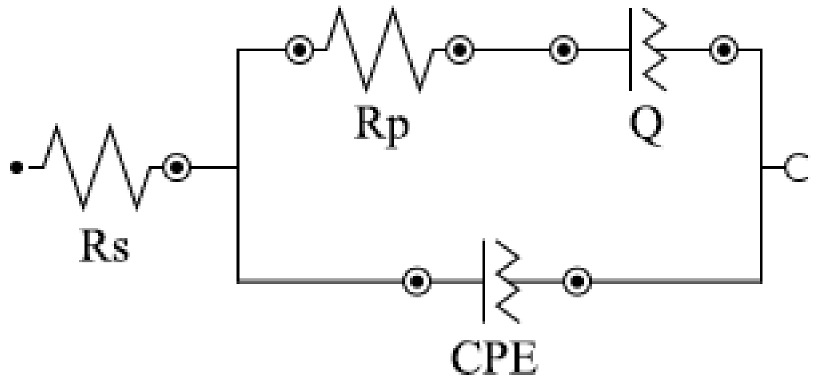

Figure S6. Their electrical equivalent circuit diagram for fitting the solid-liquid interface.

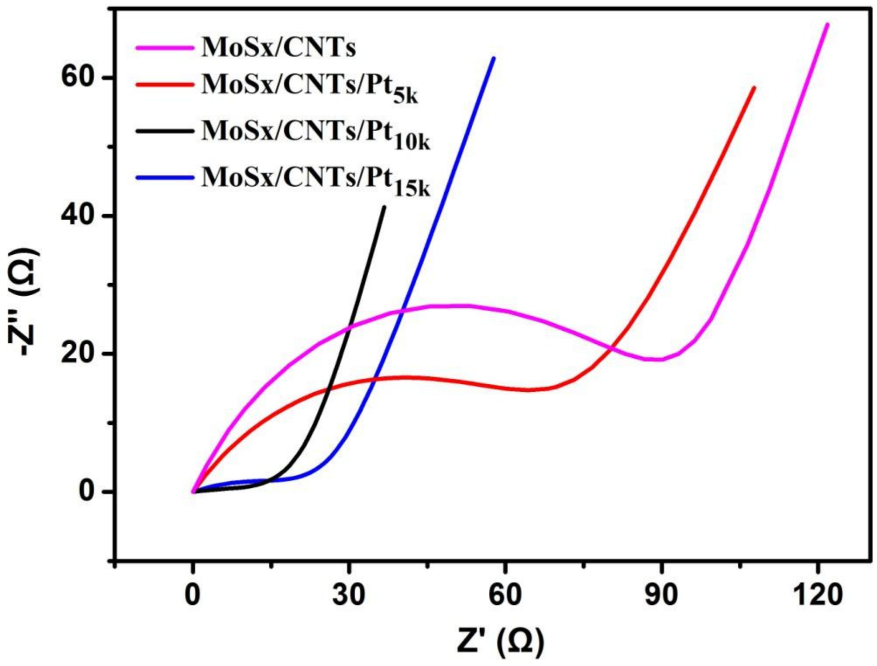

Figure S7. The impedance parameters derived by fitting the EIS on the MoSx/CNTs/Pt composites with various deposition cycles.

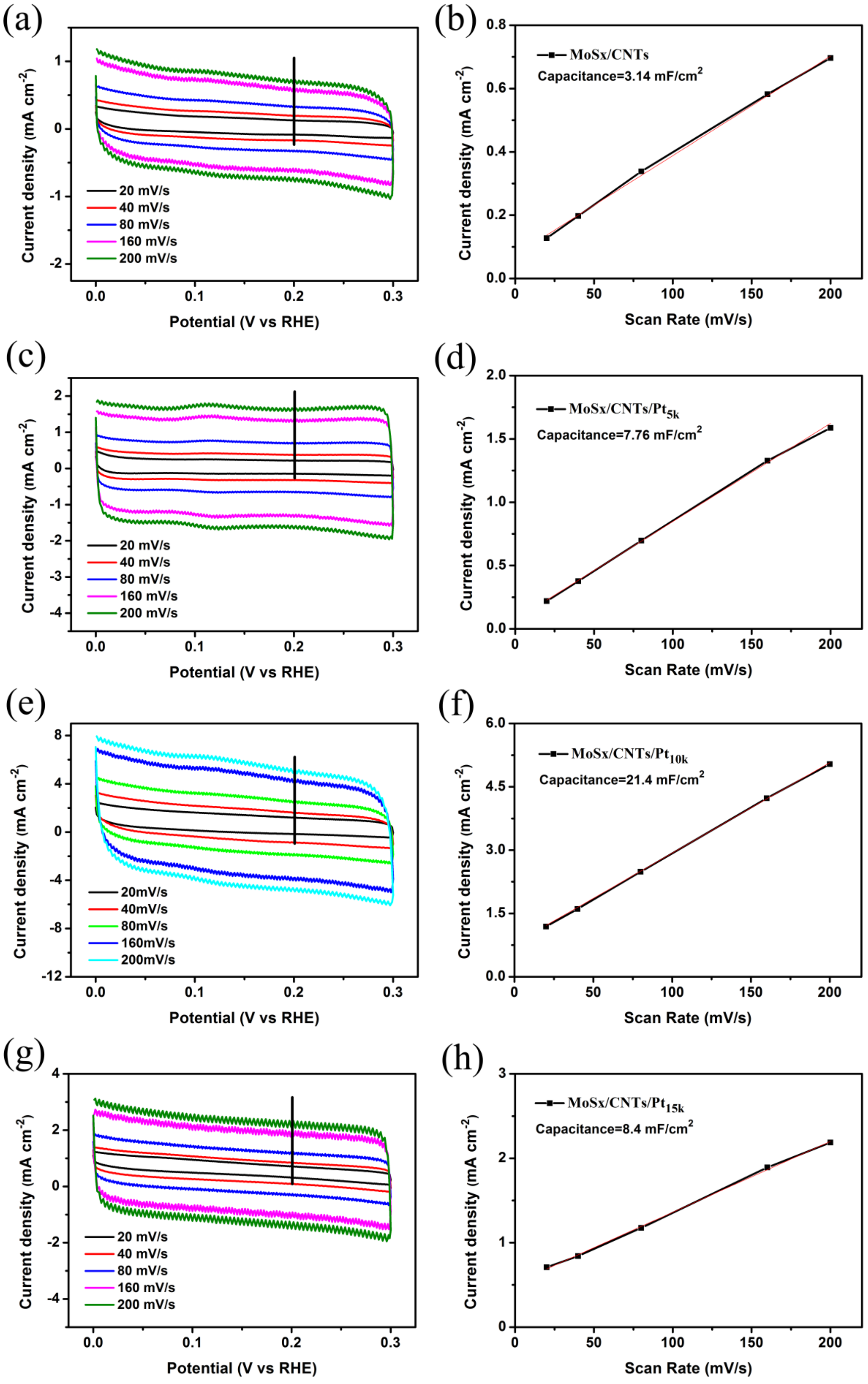

Figure S8. The standard CV curves and C_{dl} for different samples.

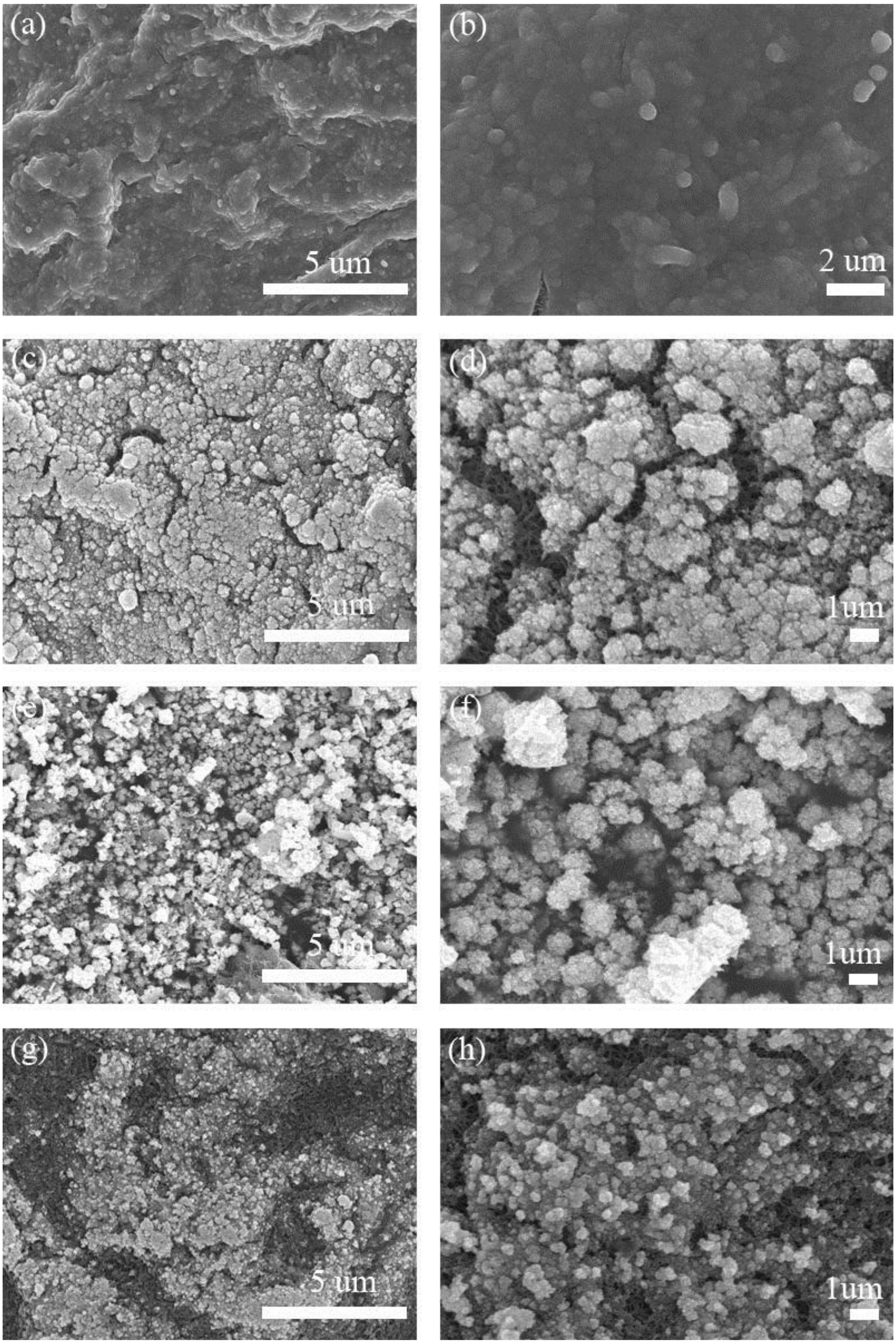

Figure S9. The various morphology of the different electrodeposition cycles a,b) SEM images of MoSx/CNTs hybrid; c,d) SEM images of MoSx/CNTs/Pt_{5k} hybrid; e,f) SEM images of MoSx/CNTs/Pt_{10k} hybrid; g,h) SEM images of MoSx/CNTs/Pt_{15k} hybrid.

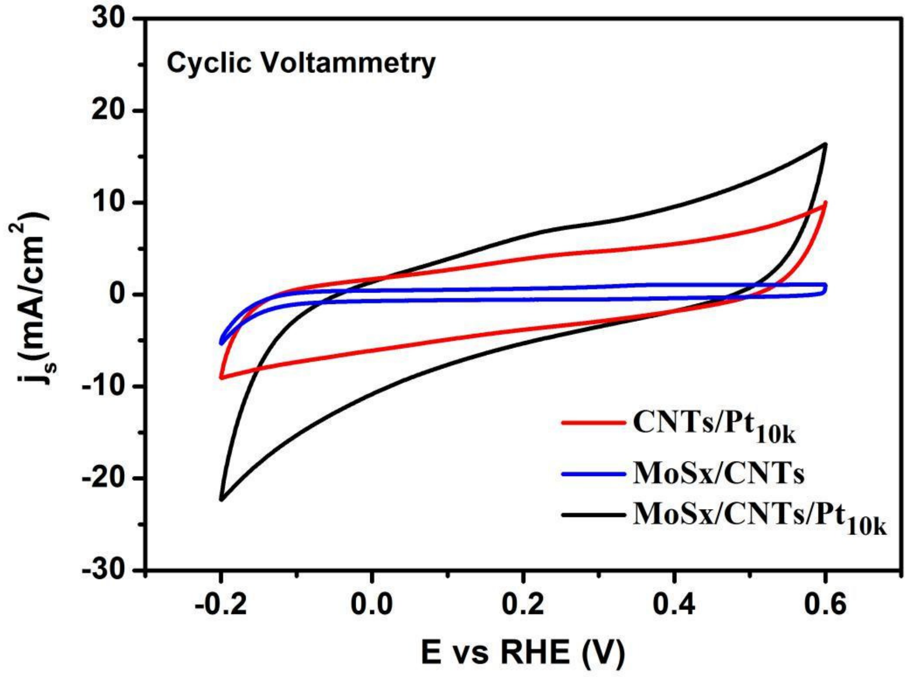

Figure S10. The cyclic voltammetrys for CNTs/Pt_{10k}, MoSx/CNTs and MoSx/CNTs/Pt_{10k} in 1.0 M PBS at a scan rate of 50 mV/s

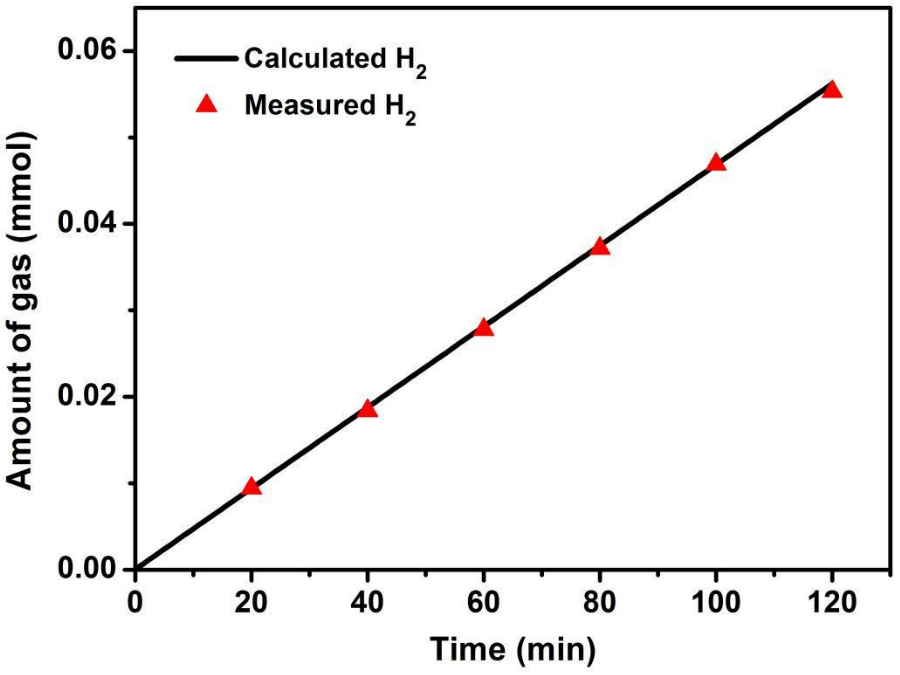

Figure S11. The amount of hydrogen theoretically calculated and experimentally measured versus time for MoSx/CNTs/Pt_{10k} in 0.5 M H_2SO_4.

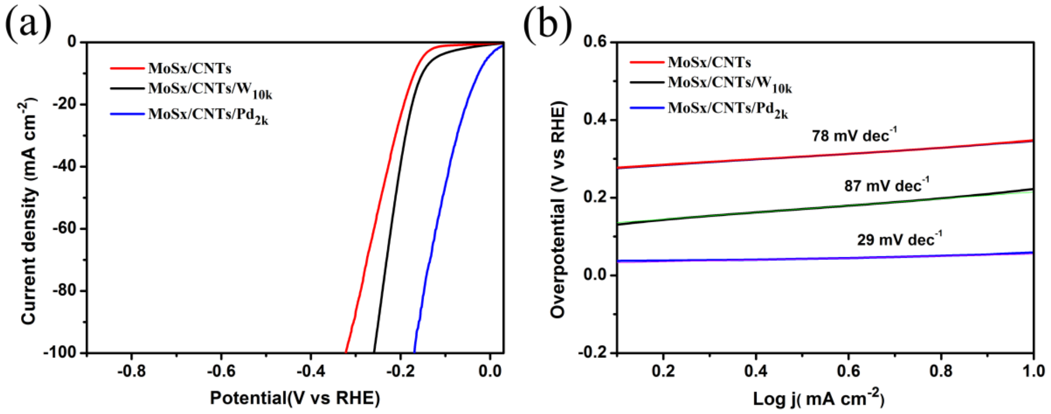

Figure S12. a) The polarization curves for MoSx/CNTs, MoSx/CNTs/W_{10k}, MoSx/CNTs/Pd_{2k} in 0.5M H_2SO_4 solution at a scan rate of 10 mV s^{-1} and b) the corresponding Tafel plots;

Table S2. The impedance parameters derived by fitting the EIS responses on the MoSx/CNTs/Pt composites with various deposition cycles in H_2SO_4

**Samples**	**RS (Ω)**	**RP (Ω)**	**CPE (μMho)**
MoSx/CNTs	327	103	4.41
MoSx/CNTs/Pt_{5k}	380	88.6	7.82
MoSx/CNTs/Pt_{10k}	385	52.0	42.2
MoSx/CNTs/Pt_{15k}	418	58.6	21.4

Table S3. Comparision of HER performance in acid media for our electrodeposition sample with other reported electrocatalysts.

Catalyst (pt loading)	Current density ($mA\ cm^{-2}$)	η at corresponding j(mV vs RHE)	Tafel slope (mV per dec)	Stability	Ref.
Pt-VGNSAs/CC ($41.92\ ug\ cm^{-2}$)	10	60	28.5	12000 s	S1
Pt−$Co(OH)_2$/CC (5.7wt%)	10	32	70	20 h	S2
Pt−MoO_2/MWCNTs (0.5wt%)	10	60	43	2000 cycles	S3
MoS_2@Pt (2.45wt%)	10	70	36	500 min	S4
NiFe LDH-Pt-ht (1.51wt%)	10	101	127	9 h	S5
Pt-Decorated SnS_2 Nanosheets (0.37wt%)	10	117	69	12 h	S6
Pt NC/N-graphene (5.0wt%)	10	30	28	9 h	S7
C-MoS_2	200	198	47	3000 cycles	S8
mPF-Co-MoS_2	10	156	74	5000 cycles	S9
$MoS_{2(1-x)}Se_{2x}/NiSe_2$	10	68	42.1	16 h	S10
P-Mo_2C@C	10	89	42	3000 cycles	S11
MoS_2/rGO	10	117	43.2	3000 cycles	S12
Sub-MoSx-CNTs	10	106	37	1400 cycles	S13

Pt-MOSx-CNTs (0.55wt%)	10	25	27	100 h	Our work
BCF/Mo_2C	20	115	84.8	50 h	S14
Pt/NCNTs (0.74wt%)	10	40	33	18000 s	S15
Er-WS_2-Pt	10	43	27	15h	S16
RuCoP	10	11	31	150h	S17
Rh/SiNW	0.1	44	24	500000s	S18
Li-PPSNDs	10	91	29	12h	S19
N-WC nanoarray	10	89	75	20h	S20
Fe/GD	10	66	37.8	60h	S21
SWCNTs/$MoSe_2$	10	100	63	40000s	S22
MoP/CNT	10	83	60	40h	S23
CoSx Silk-Cocoon	10	42	41	20000s	S24
Cu_7S_4@MoS_2	10	133	48	35000s	S25
CoP@PS/NCNT	10	80	53	250min	S26
Co:WS_2/Co:$W_{18}O_{49}$	10	210	49	1000cycles	S27
3D $Co_{1-x}V_X$SP	10	55	50	6h	S28

References:

[S1] H. Zhang, W. Ren, C. Guan, C. Cheng, J. Mater. Chem. A **2017**, 5, 22004.

[S2] Z. Xing, C. Han, D. Wang, Q. Li , X. Yang, ACS Catal. **2017**, 7, 7131.

[S3] X. Xie, Y.-F. Jiang, C.-Z. Yuan, N. Jiang, S.-J. Zhao, L. Jia & A.-W. Xu, J.Phys. Chem. C **2017**, 121, 24979.

[S4] X. Y. Xu, X. F. Dong, Z. J. Bao, R. Wang, J. G. Hu , H. B. Zeng, J. Mater. Chem. A **2017**, 5, 22654.

[S5] S. Anantharaj, K. Karthick, M. Venkatesh, T. V. S. V. Simha, A. S. Salunke, L. Ma, H. Liang , S. Kundu, Nano Energy **2017**, 39, 30.

[S6] G. Liu, Y. Qiu, Z. Wang, J. Zhang, X. Chen, M. Dai, D. Jia, Y. Zhou, Z. Li, P. Hu, ACS Appl. Mater. Interfaces **2017**, 9, 37750.

[S7] B. Jiang, F. Liao, Y. Sun, Y. Cheng, M. Shao, Nanoscale **2017**, 9, 10138.

[S8] S. Dou, J. Wu, L. Tao, A. Shen, J. Huo, S. Wang. Nanotechnology **2016**, 27, 045402.

[S9] J. Deng, H. Li, S. Wang, D. Ding, M. Chen, C. Liu, Z. Tian, K. S. Novoselov, C. Ma, D. Deng , X. Bao, Nat. Commun. **2017**, 8, 14430.

[S10] H. Zhou, F. Yu, Y. Huang, J. Sun, Z. Zhu, R. J. Nielsen, R. He, J. Bao, W. A. Goddard, III, S. Chen, Z. Ren, Nat. Commun. **2016**, 7, 12765.

[S11] Z. Shi, K. Nie, Z.-J. Shao, B. Gao, H. Lin, H. Zhang, B. Liu, Y. Wang, Y. Zhang, X. Sun, X.-M. Cao, P. Hu, Q. Gao , Y. Tang, Energy Environ. Sci. **2017**, 10, 1262.

[S12] Y. Sun, F. Alimohammadi, D. Zhang, G. Guo. Nano Lett. **2017** , 17, 1963.

[S13] P. Li, Z. Yang, J. Shen, H. Nie, Q. Cai, L. Li, M. Ge, C. Gu, X. Chen, K. Yang, L. Zhang, Y. Chen, S. Huang, ACS Appl. Mater. Interfaces **2016**, 8, 3543.

[S14] J. Xiao, Y. Zhang, Z. Zhang, Q. Lv, F. Jing, K. Chi, S. Wang, ACS Appl.Mater. Interfaces **2017**, 9, 22604.

[S15] R. Ma, Y. Zhou, F. Wang, K. Yan, Q. Liu , J. Wang, Materials Today Energy **2017**, 6, 173.

[S16] L. Ji, J. Wang, S. Zuo, Z. Chen, J. Phys. Chem. C **2017**, 121, 8923.

[S17] T. Chao, X. Luo, W. Chen, B. Jiang, J. Ge, Y. Lin, G. Wu, X. Wang, Y. Hu, Z. Zhuang, Y. Wu, X. Hong , Y. Li, Angew. Chem. Int. Ed . **2017**, 56, 16047.

[S18] S. Bai, C. Wang, M. Deng, M. Gong, Y. Bai, J. Jiang , Y. Xiong. Angew. Chem. Int. Ed.

2014, 53, 12120.

[S19] X. Zhang, Z. Luo, P. Yu, Y. Cai, Y. Du, D. Wu, S. Gao, C. Tan, Z. Li, M. Ren, T. Osipowicz, S. Chen, Z. Jiang, J. Li, Y. Huang, J. Yang, Y. Chen, C. Y. Ang, Y. Zhao, P. Wang, L. Song, X. Wu, Z. Liu, A. Borgna, H. Zhang, Nat. Catal. **2018**, 1, 460.

[S20] N. Han, K. R. Yang, Z. Lu, Y. Li, W. Xu, T. Gao, Z. Cai, Y. Zhang, V. S. Batista, W. Liu, X. Sun, Nat. Commun. **2018**, 9, 924.

[S21] Y. Xue, B. Huang, Y. Yi, Y. Guo, Z. Zuo, Y. Li, Z. Jia, H. Liu, Y. Li, Nat .Commun .**2018**, 9, 1460.

[S22] L. Najafi, S. Bellani, R. Oropesa-Nuñez, A. Ansaldo, M. Prato, A. E. Del Rio Castillo, F. Bonaccorso, Adv. Energy Mater. **2018**, 8, 1703212.

[S23] X. Zhang, X. Yu, L. Zhang, F. Zhou, Y. Liang, R. Wang. Adv. Funct. Mater. **2018**, 28, 1706523.

[S24] C. Wang, T. Wang, J. Liu, Y. Zhou, D. Yu, J.-K. cheng, F. Han, Q. Li, J. Chen, Y. Huang, Energy Environ. Sci. **2018**, 39, 948.

[S25] J. Xu, J. Cui, C. Guo, Z. Zhao, R. Jiang, S. Xu, Z. Zhuang, Y. Huang, L. Wang, Y. Li, Angew. Chem. Int. Ed. **2016**, 55, 6502.

[S26] D. J. Li, J. Kang, H. J. Lee, D. S. Choi, S. H. Koo, B. Han, S. O. Kim, Adv. Energy Mater. **2018**, 8, 1702806.

[S27] X. Shi, M. Fields, J. Park, J. M. McEnaney, H. Yan, Y. Zhang, C. Tsai, T. F. Jaramillo, R. Sinclair, J. K. Nørskov, X. Zheng, Energy Environ. Sci. **2018**, 39, 1111.

[S28] N. Q. Tran, V. Q. Bui, H. M. Le, Y. Kawazoe, H. Lee. Adv. Energy Mater. **2018**, 8, 1702139.
